# Lipid Nanoparticles as Delivery Systems for RNA-Based Vaccines

**DOI:** 10.3390/pharmaceutics13020206

**Published:** 2021-02-02

**Authors:** Basmah N. Aldosari, Iman M. Alfagih, Alanood S. Almurshedi

**Affiliations:** Department of Pharmaceutics, College of Pharmacy, King Saud University, Riyadh 11495, Saudi Arabia; baldosari@ksu.edu.sa (B.N.A.); marshady@ksu.edu.sa (A.S.A.)

**Keywords:** lipid nanoparticles, cationic lipids, vaccines, cancer immunotherapy, mRNA, nucleic acid, adjuvant, delivery system, nanotechnology

## Abstract

There has been increased interest in the development of RNA-based vaccines for protection against various infectious diseases and also for cancer immunotherapies. Rapid and cost-effective manufacturing methods in addition to potent immune responses observed in preclinical and clinical studies have made mRNA-based vaccines promising alternatives to conventional vaccine technologies. However, efficient delivery of these vaccines requires that the mRNA be protected against extracellular degradation. Lipid nanoparticles (LNPs) have been extensively studied as non-viral vectors for the delivery of mRNA to target cells because of their relatively easy and scalable manufacturing processes. This review highlights key advances in the development of LNPs and reviews the application of mRNA-based vaccines formulated in LNPs for use against infectious diseases and cancer.

## 1. Introduction

Vaccination is considered an effective approach in controlling infectious diseases. Conventional vaccines based on live attenuated pathogens are known to activate both humoral and cellular immunity. However, this type of vaccine suffers from safety concerns due to the risk of the attenuated pathogen reverting to a pathogenic form that can induce infection. On the other hand, subunit vaccines are recognized as safe alternatives to live attenuated vaccines but are less efficient at inducing the cellular immunity needed to eliminate intracellular pathogens [1].

New vaccine technologies based on viral vectors and nucleic acids, such as plasmid DNA and mRNA, are capable of inducing humoral and cytotoxic T cell immunity responses based on the expression of vaccine antigens in situ. This property assists in eliminating the possibility of intracellular infections while achieving protective effects [2].

Although plasmid DNA vaccines have been demonstrated to be safe and effective in human clinical trials, the delivery of plasmid DNA into the nucleus remains inadequate. Therefore, research into mRNA-based vaccines has been initiated because this type of vaccine can be delivered for antigen expression without any requirement to cross the membrane barrier of the nucleus [3,4]. If mRNA is transported across the membrane of the nucleus, it does not integrate with or modify the host cell genome [5]. In addition, the physicochemical properties of mRNA, such as structural, binding, and translational properties, remain unaffected upon the encoding of multiple proteins with various physical and chemical properties. Compared to other types of vaccines, mRNA-based vaccines can be easily and rapidly produced at low cost once information on the gene sequence of the infectious pathogen is obtained [3,4].

Generally, mRNA vaccines are divided into two types: Non-replicating or conventional type and the self-amplifying type. The non-replicating type is structurally simple with a small RNA molecule. However, its in vivo stability and activity are limited by the limited duration of antigen expression inside cells. The low antigen expression typical of this type of mRNA vaccine can be increased by optimizing the delivery formulation [2].

Chemical modification and sequence optimization of the nucleoside base of conventional mRNA can be used to increase protein expression and immunogenicity. Comparison of the efficacy of sequence-optimized mRNA to its nucleoside-modified counterpart revealed unclear results that were attributed to differences in various parameters such as the sequence optimization algorithms, type of modified nucleosides, route of administration, and other experimental conditions [6,7]. However, it was reported that utilization of lipid nanoparticles (LNPs) to deliver either sequence-optimized or nucleoside-modified mRNA resulted in strong activation of innate immunity in addition to increased infiltration of neutrophils and dendritic cells to the site of injection and draining lymph nodes after immunization of rhesus macaques [8] and mice [9].

The ability of self-amplifying mRNA to encode multiple antigens was evaluated by Magini et al. [10] using the nucleoprotein and M1 proteins of influenza virus, and by Brito et al. [3] using the Hg/gL protein complex of human cytomegalovirus (HCMV). Potent T cell responses in addition to protection against viral infection were observed following the immunization of animals. The high levels of antigen expression produced by self-amplifying mRNA are due to their ability to self-amplify within cells [2]. Both non-replicating and self-amplifying mRNA-based vaccines can be employed for prophylaxis against infectious diseases [11].

The stability of mRNA under physiological conditions represents a major challenge for efficient intracellular delivery of mRNA-based vaccines. In designing this type of vaccine, the high susceptibility of mRNA toward hydrolysis by omnipresent ribonuclease enzymes should be taken into consideration [12]. Various strategies have been utilized to deliver mRNA. For example, the formation of RNA conjugates may protect the nucleic acid against degradation but, at the same time, it may enhance binding to serum proteins and subsequently lead to aggregation and vascular blockage [13].

On the other hand, utilization of viral vectors for the delivery of nucleic acids has been associated with safety drawbacks, such as the possibilities of excessive viral replication in immunosuppressed patients and immunodominance of viral antigens over vaccine antigens [14] in addition to manufacturing difficulties [15] when rapid and large-scale production is needed. Therefore, nonviral vectors are preferred as vehicles for the delivery of mRNA, especially with the wide variety of materials that can be used in the design of these vectors in addition to various formulation techniques that can be adopted for their manufacture [16,17,18]. Compared to viral vectors, nonviral carriers are less immunogenic and exhibit lower transfection efficiency. In addition, these vectors are easier to manufacture and capable of carrying larger payloads of genetic material [1]. Different materials, such as peptides [19], polymers [20], nanoparticles [21], and lipids [22], can be used for the delivery of nonviral vectors.

**Figure 1:**
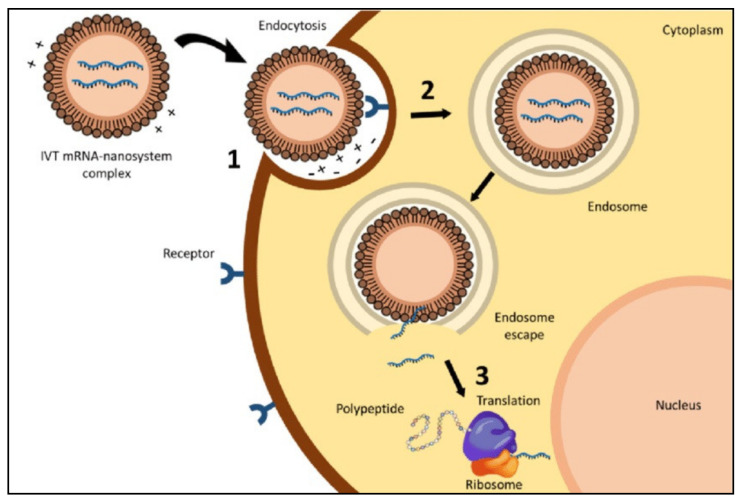
Intracellular barriers for in vitro transcribed (IVT) mRNA delivery: (1) interaction between the delivery system and the cell membrane, (2) endocytosis, and (3) endosomal escape and release of the mRNA to start the translation process (reproduced from Gomez-Aguado et al. [23]).

Efficient binding of mRNA to nonviral vectors may protect the nucleic acid against degradation in the extracellular space and assist in ensuring its localization at the targeted cellular membrane. The delivery of the nucleic acid requires cellular uptake of the delivery system followed by endosomal escape of the nucleic acid into the cytosol of the targeted cell [1,23]. For this process to take place, a suitable delivery system is crucially required to overcome different barriers as presented in Figure 1.

Lipid nanoparticles are among the most widely investigated nonviral vectors for the in vivo delivery of nucleic acid vaccines [24]. Support for the use of LNPs in the systemic delivery of short interfering RNA (siRNA) was provided in 2018 with the Food and Drug Administration (FDA)’s approval of Onpattro^®^ (patisiran) for treatment of polyneuropathy caused by amyloidosis. LNPs are also being increasingly used in the field of gene therapy, protein replacement therapy, and mRNA-based vaccines developed against both cancer and infectious diseases [25].

In one study [26], the transfection efficiencies of gene carrier-mediated mRNA were evaluated and compared after in vitro and in vivo administration of naked mRNA (dissolved in Ringer’s Lactate) and mRNA nanoparticles formulated in commercial lipid-based transfection agent. This study demonstrated that human and mouse dendritic cells (DCs) can be efficiently transfected using lipid-based formulations of mRNA. The authors also demonstrated that in vivo transfection efficiency produced by lipid-based formulations of mRNA varied according to the route of administration.

Table 1 summarizes some of the work conducted to investigate the preclinical efficacy of mRNA vaccines against infectious diseases and cancer when prepared in LNPs or other lipid-based formulations. LNPs to be used particularly in the design of RNA-based vaccines require specific types and proportions of lipid components in addition to special manufacturing procedures.

The present work reviews the components used for designing LNPs for the purpose of delivery of mRNA-based vaccines and outlines different methods for their production in addition to factors that contribute to the efficacy and uptake of this class of vaccines. In addition, pre-clinical and clinical trials conducted to investigate the potential application of mRNA-based vaccines developed as LNPs against infectious diseases and cancer will be highlighted.

## 2. Overview of Various Lipid-Based Formulations for the Delivery of Nucleic Acids

**Figure 2:**
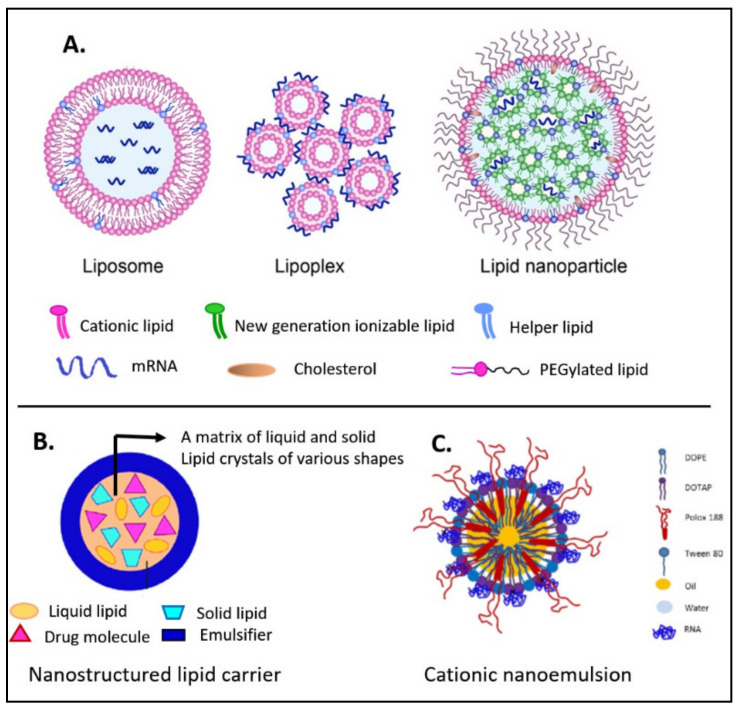
Key lipid nanocarriers of mRNA: (**A**) liposome, lipoplex, and lipid nanoparticle; (**B**) nanostructured lipid carrier; (**C**) cationic nanoemulsion (reproduced and modified from Granot et al. [53]).

Different lipids have been commonly used to fabricate various lipid-based formulations for the delivery of nucleic acids [48]. Traditional liposomes, lipoplexes, cationic nanoemulsions (CNEs), and nanostructured lipid carriers (NLCs) were developed as delivery systems for nucleic acids. In addition, more advanced delivery systems of LNPs have emerged and become more effective for delivering nucleic acids compared to the classical lipid-based formulations (Figure 2). These advanced LNPs may not show a lipid bilayer enclosing an aqueous core. Instead, they may present a micelle-like structure that encapsulates drug molecules inside a non-aqueous core. In addition, LNPs do not exhibit electrostatic complexation with their nucleic acid contents [25].

### 2.1. Liposomes

Liposomes are spherical vesicles comprising unilamellar or multilamellar phospholipid bilayers enclosing an aqueous core in which the drug of choice can be encapsulated. They are prepared from materials possessing polar head (hydrophilic) groups and nonpolar tail (hydrophobic) groups (Figure 2). The interaction between these groups induces the formation of vesicles [49]. Liposomes are commonly used as drug carriers because of their biodegradability, efficacy, minimal toxicity, and ease of formulation. In the field of delivery of mRNA-based vaccines, liposomes were found to be promising in infectious diseases [34] as well as in cancer immunotherapy [50]. For example, one study demonstrated that intratumoral injection of mRNA–liposomal complexes was highly effective and comparable to the corresponding DNA–liposomes in achieving in situ tumor transfection [51]. Later on, Zhou et al. [52] developed neutral liposomes of mRNA vaccine encoded with the human melanoma antigen glycoprotein 100 (gp100). Direct injection of the developed liposomes in the spleen of mice resulted in the suppression of tumor growth and significant survival prolongation compared to the control group [52].

Cationic lipids employed in formulating liposomes designed for the delivery of nucleic acids are amphiphilic in nature and consist of a positively charged (cationic) amine head group linked to a hydrocarbon chain or cholesterol derivative via glycerol. An important property of these lipids is the ability of their positively charged head group to undergo electrostatic interaction with the negatively charged nucleic acids, permitting the encapsulation of the nucleic acid in the core of the lipid-based nanoparticles [53]. Early reports showed that using cationic lipids such as N-[1-(2,3-dioleyloxy) propyl]-N,N,N-trimethylammonium chloride (DOTMA) in the preparation of liposomes (lipofectin) for transfection of mRNA into mouse cells resulted in a highly effective transfection system for the nucleic acid [54,55].

Cationic lipids employed for mRNA-based vaccines allow encapsulation of mRNA and also act as immunogenic agents [53]. For instance, a potent immune response was observed after subcutaneous injection of mice with mRNA complexed with the cationic lipid 1,2-dioleoyl-3-trimethylammonium propane and 1,2-dioleoyl-sn-glycero-3-phosphoethanolamine (DOTAP/DOPE) that encoded the human immunodeficiency virus (HIV)-1 Gag antigen. The observed potent immune responses led to specific killing of Gag peptide-pulsed cells and gave rise to humoral responses [56]. On the other hand, complexing mRNA with liposomes based on Genzyme lipid 67 (GL67) did not produce significant expression of luciferase in murine lungs after intrapulmonary administration. By contrast, administration of pDNA–GL67 liposomes produced detectable luciferase expression in the lungs of mice. These differences were attributed to the limited stability of the mRNA–GL67 liposomes in biological fluids [57].

In addition, a range of cationic liposomes, especially those based on 1,2-dioleoyl-3-trimethylammonium propane (DOTAP), was proposed to act as vaccine adjuvants. These types of cationic liposomes perform as immunomodulators that stimulate the innate immune response in an antigen (or pathogen)-independent manner [58,59]. The immunostimulatory effects of cationic liposomes were found to be related to the nature of the amine head group, fluidity of the lipid, or degree of acyl chain saturation. It was proposed that lipids with quaternary amine head groups are more effective immunostimulators than lipids containing tertiary amines. Moreover, lipids having unsaturated acyl chains or short saturated chains stimulate the release of pro-inflammatory mediators and cytokines more than lipids possessing long saturated acyl chains [59]. Therefore, the potential immune toxicity of cationic liposomes that are employed as delivery systems for nucleic acids must be carefully evaluated.

### 2.2. Lipoplexes

Lipoplexes are liposome-based formulations that form upon electrostatic interaction of cationic liposomes with RNAs. Formed lipoplexes possess distinct internal arrangements of molecules (Figure 2) that arise due to the transformation from liposomal structure into compact RNA–lipoplexes [48]. These formulations are characterized by their poor encapsulation of the nucleic acid and poor tolerability and, therefore, have been excluded from clinical studies. In addition, rapid removal of lipoplexes from blood circulation and enhanced immune response may be encountered after intravenous administration due to the fact of their positively charged nature, tendency to aggregate and incomplete entrapment of the nucleic acid [25].

### 2.3. Cationic Nanoemulsions

Cationic lipids can also be used to formulate CNEs (Figure 2). Brito et al. [38] developed a cationic oil-in-water nanoemulsions in which the oil phase comprises DOTAP (as a cationic lipid) and squalene (as a safe and potent adjuvant). Nano-sized droplets were formed when the oil phase was added to the aqueous phase and stabilized by hydrophilic (Tween 80) and hydrophobic (sorbitan trioleate) surfactants. The prepared CNE was used to bind self-amplifying mRNA vaccine encoding the antigens of respiratory syncytial virus (RSV), HIV, and human cytomegalovirus. A potent immune response was observed in mice after two intramuscular injections of relatively low doses of the CNE-formulated self-amplifying RNA vaccine. In addition, HIV neutralizing antibodies were generated at high levels in rabbits, and induced T cell responses were observed in rhesus macaques following intramuscular injections of self-amplifying RNA vaccine expressing human cytomegalovirus envelope glycoprotein B [38].

### 2.4. Nanostructured Lipid Carriers

Nanostructured lipid carriers (NLCs) are among the lipid-based formulations that can be used for the delivery of mRNA-based vaccines (Figure 2). NLCs are considered a hybrid formulation between solid lipid nanoparticles and oil-in-water emulsions. The core of NLCs is a mixture of solid and liquid lipids. This semi-crystalline matrix provides the formulation with better colloidal stability depending on the amount of solid lipid used. Compared to other solid lipid nanoparticles, NLCs are easily produced and sterilized and possess low toxicity [23]. A An NLC formulation was developed and combined with self-amplifying mRNA encoding Zika virus antigens in one study, resulting in100% protection against Zika virus in mice following a single intramuscular dose as low as 10 ng [43].

## 3. Composition of LNPs

**Figure 3:**
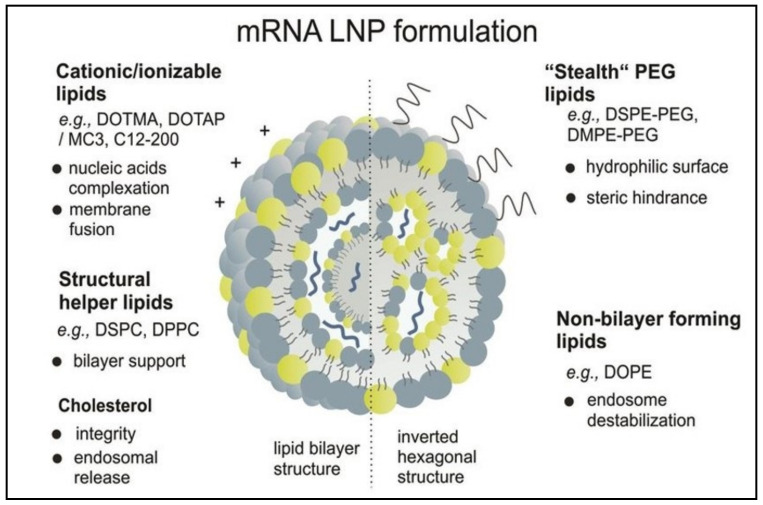
Schematic representation of mRNA lipid nanoparticles (reproduced from Sempler et al. [61]). DOTMA: 1,2-di-O-octadecenyl-3-trimethylammonium propane; DOTAP: 1,2-dioleoyl-3-trimethylammonium-propane; MC3: D-Lin-MC3-DMA; DSPC: 1,2-distearoyl-sn-glycero-3-phosphocholine; DPPC: 1,2-dipalmitoyl-sn-glycero-3-phosphocholine; DSPE-PEG: 1,2-distearoyl-sn-glycero-3-phosphoethanolamine-Poly(ethylene glycol; DMPE-PEG: 1,2-dimyristoyl-sn-glycero-3-phosphoethanolamine polyethylene glycol; DOPE: 1,2-dioleoyl-sn-glycero-3-phosphoethanolamine.

LNPs used for RNA-based vaccines should be prepared from precise proportions of specific lipid components following specialized manufacturing procedures. As presented in Figure 3, LNPs typically consist of a cationic/ionizable lipid and helper lipids such as phospholipids, cholesterol, and/or PEGylated lipids [1,25,49,60,61].

### 3.1. Ionizable Cationic Lipids

The use of cationic lipids in the development of traditional lipid formulations resulted in a high encapsulation efficiency of nucleic acids because of the electrostatic interactions between the positively charged lipid and the negatively charged nucleic acid. However, intravenous administration of these highly charged lipid formulations may lead to rapid elimination from the blood in addition to stimulation of the immune system. These poor tolerability problems have led to the utilization of emerging ionizable cationic lipids as a critical component in the development of more enhanced LNPs [25].

Ionizable cationic lipids were first introduced to the field of delivery of nucleic acids by Semple et al. [62]. These lipids are characterized by their ability to change their charge depending on the pH of the environment such that they acquire a positive charge at acidic pH and become neutral at physiological pH. The acquired positive charge enables the lipid to encapsulate an RNA molecule and to interact with the endosomal membrane during the fusion process and to then release the nucleic acid into the cytosol. On the other hand, the neutral charge gained by these lipids at a physiological pH prevents rapid removal of the lipid formulation from the blood, and thus, the tolerability and pharmacokinetic properties are improved [25,53]. Consequently, the lipid formulation exhibits an extended half-life in circulation (approximately 30 min or more) following intravenous administration in addition to enhanced accumulation in diseased tissues, such as liver and solid tumors. Ionizable lipids show an improved tolerability profile compared to cationic lipids, which are more likely to cause cellular toxicity. In addition, particles containing cationic lipids are more prone to aggregating and depositing in fine capillary beds, leading to undesirable effects [25].

Different structural modifications were carried out on ionizable lipids to enhance their potency in delivering nucleic acids [63,64]. For instance, a dramatic increase in the potency of ionizable lipids was obtained by increasing the degree of unsaturation in their hydrophobic domain. This was explained on the basis that increasing the number of double bonds may promote the formation of fusogenic groups in nanoparticles, which may enhance the fusion process and endosomal delivery [63]. In addition, it was reported that the potency of the formulation is strongly affected by its acid dissociation constant (pKa), which corresponds to the pH at which the ionizable lipid or the LNPs become positively charged. Therefore, it is important that the nanoparticles possess a neutral surface at a physiological pH and become charged after their cellular uptake into the acidic environment of the endosomes [64]. Further enhancement of the LNP potency in delivering nucleic acids can be achieved by monitoring the LNP compositions. For example, a marked increase in the content of ionizable lipids (up to 57%) and a reduction in the polyethylene glycol (PEG) content (from 10% to 1.4%) resulted in a dramatic increase in the potency of the lipid formulation [64]. Similar compositions of 50% ionizable lipids and 1.5% PEG are still employed in the development of different mRNA-based formulations [25].

The high biodegradability properties of ionizable lipids employed in formulating LNPs, especially those intended for frequent administration, should also be considered to avoid toxicity mediated by lipids. These properties can be ascertained in the lipids by introducing carboxylic ester groups into the hydrophobic lipid tails. In vivo cleavage of these groups by esterase enzymes may assist in metabolizing the compounds into more water-soluble products that can be easily eliminated from plasma and tissues within a few hours [65].

Utilization of ionizable lipids to formulate LNPs for RNA vaccines has been documented in various research studies [30,44,66]. For instance, LNPs prepared with an ionizable cationic lipid (that is proprietary to Acuitas) in addition to phosphatidylcholine, cholesterol, and PEG–lipid were used to encapsulate mRNA encoding the pre-membrane and envelop glycoproteins of a strain of Zika virus. Intra-dermal immunization of mice and non-human primates with a single dose of these LNP formulated-mRNA induced protective efficacy in both [30].

Another group of researchers developed an LNP formulation to deliver mRNA vaccines that would induce a cytotoxic T cell response in B16F10 melanoma-bearing mice [44]. The LNPs formulated with ionizable cationic lipid, phospholipid, cholesterol, and PEG–lipid were used to entrap modified mRNA coding for the tumor-associated antigens gp100 and TRP2. Subcutaneous immunization using the formulated LNPs resulted in strong T cell activation with subsequent shrinkage of tumor size and prolonged survival of the treated mice. The study also showed that the addition of lipopolysaccharide (LPS) as an adjuvant to the optimized LNP formulation enhanced the potency of the mRNA vaccine and improved the survival of mice that received these adjuvant-containing LNPs compared to the control group treated with the LNPs formulated without the adjuvant.

In order to enhance the in vivo potency of LNPs for the delivery of RNA, newly developed ionizable lipids were generated, termed “lipidoids”. In a study by Chahal et al. [36], modified dendrimer nanoparticles developed using a dendrimer-based lipidoid and a PEG–lipid were used to encapsulate self-amplified mRNA encoding the hemagglutinin (HA) protein of an H1N1 influenza virus, the glycoprotein (gp) of Ebola virus, or the six *Toxoplasma gondii*—specific antigens (apical membrane antigen 1 (AMA1), Dense granule protein 6 (GRA6), surface antigen 1 (SAG1), surface antigen 2A (SAG2A), rhoptry protein 2A (ROP2A), and rhoptry protein 18 (ROP18)). Two intramuscular vaccinations of mice with the formulated mRNA vaccine encoding the glycoprotein of the Ebola virus resulted in high gp antibody titers in all treated mice with complete protection against the Ebola virus. Similarly, the vaccine encoding HA of the H1N1 influenza virus or the antigen of *Toxoplasma gondii* protected all mice against the pathogens of these infections after a single intramuscular injection. The same authors used the same dendrimer-based lipidoid nanoparticles in a different study [42] to develop an mRNA vaccine against Zika virus.

### 3.2. Helper Lipids

Helper lipids, such as PEGylated lipids, cholesterol, and phosphatidylcholine, possess distinct functional properties and are usually included in mRNA–LNP formulations to enhance the stability of nanoparticles [61]. Helper lipids also promote the cellular uptake and destabilization of the lipid bilayer, and thereby, improve the efficiency of nucleic acid delivery [67].

#### 3.2.1. PEG–Lipids

PEGylated lipids, or PEG–lipids, represent important components that are usually located on the surface of the LNPs. PEG–lipids consist of a hydrophilic molecule of PEG conjugated to a hydrophobic alkyl (or lipid) chain, where the PEG domain is attached to the LNP surface while the alkyl chain is attached to the LNP bilayer. The incorporation of PEGylated lipids in LNPs is known to extend the circulation time of LNPs due to their steric barrier effect. This property reduces binding of LNPs to plasma proteins (opsonins), reduces rapid elimination by the reticuloendothelial system, and consequently, assists in the accumulation of the nanoparticles at disease sites [25,49]. In addition, the steric barrier properties of PEGylated lipids may prevent fusion or aggregation of nanoparticles during manufacturing, and this would result in a homogenous formulation with a small particle size (50–100 nm) and narrow polydispersity index [25].

The amount of PEGylated lipids incorporated in LNPs should be controlled carefully and kept to a minimum. Having higher PEG contents usually increases the residence time of LNPs in the blood circulation but may prevent their fusion with the endosomal bilayer and hinder the intracellular delivery of nucleic acids [68,69]. Therefore, a reduction in PEG content from 10% to about 1.5% (along with increasing the ionizable lipids to 57%) produced a marked improvement in the potency of mRNA-based LNPs as reported by Semple et al. [64]. Moreover, monitoring the size of PEG–lipids attached to the LNPs can be used to control the rate at which the PEG–lipids diffuse away from the nanoparticles and affect the residence time in blood circulation. Larger lipid anchors are usually used in formulating liposomes that circulate for longer duration in the blood to deliver chemotherapeutic drugs [70]. On the other hand, smaller lipid anchors are used in developing LNPs that favor shorter residence times to deliver siRNA to hepatocytes [71].

Controlling the circulation time of LNPs is important to avoid toxicity. A longer circulation time of the bound PEG–lipid may promote immunogenicity and antibody response (against the surface PEG) of the LNPs enclosing nucleic acids. A strong antibody response may accelerate removal of the formulation from blood circulation, and repeated administration may initiate acute hypersensitivity [72,73].

Anti-PEG antibodies are known to develop in patients who have been treated with PEGylated drugs or consumed PEG-containing products. These antibodies develop following repeated intravenous administration of LNPs and bind specifically to the PEG portion of the formulation, resulting in accelerated clearance of the administered LNP formulation, acute hypersensitivity reactions, and reduced drug efficacy. This phenomenon is common in immunotherapy applications that require multiple dosing for long-lasting protection. Modification of PEG molecules to be less immunogenic or the use of different administration routes may provide possible solutions to overcome this antibody response issue [1].

#### 3.2.2. Cholesterol

Incorporation of cholesterol into LNPs encapsulating mRNA or siRNA may contribute to the stability, structural integrity, and fusogenicity of LNPs [74]. Some studies have shown that the inclusion of cholesterol in LNPs resulted in high-efficiency delivery of the encapsulated DNA, probably due to enhanced membrane fusion. These improvements were explained on the basis that cholesterol exhibits low solubility in the core of nanoparticles and, therefore, may accumulate in a crystalline form on the surface of LNPs, and this may destabilize the lipid bilayer and enhance endosomal escape [75,76]. Additionally, it was recently shown that incorporation of a cholesterol analogue, like C-24 alkyl phytosterol, into LNPs has led to a remarkable enhancement in in vitro mRNA-based gene transfection [74].

#### 3.2.3. Phosphatidylcholines

Phosphatidylcholines are responsible for the formation and disruption of the lipid bilayer (because of their cylindrical geometry), and thus, may facilitate endosomal escape [1,67]. Employment of saturated, high melting point phosphatidylcholines, such as distearoylphosphatidylcholine (DSPC), may result in the production of highly stable LNPs. However, the resulting LNPs will not facilitate endosomal escape and, hence, will adversely affect the efficiency of delivering the encapsulated nucleic acids. On the other hand, utilization of unsaturated, low melting point phosphatidylcholines, like dioleoylphosphatidylcholine (DOPC), may produce fluidized LNPs that are highly susceptible to opsonization by serum protein. Therefore, phosphatidylcholines are usually combined with cholesterol to formulate highly stable LNPs that maintain their structural integrity at physiological temperatures and possess the ability to facilitate endosomal release [67].

## 4. Methods of Production of LNPs for RNA-Based Vaccines

Formerly, the most common method to formulate LNPs that are loaded with RNAs was based on hydration of the lipid components with a buffer solution containing the nucleic acid. The resulting mixture was then homogenized by means of extrusion through polycarbonate filters to produce LNPs of a smaller size. This method suffered from low encapsulation efficiencies of nucleic acid in addition to difficulties in scaling up of the process because of the highly specialized equipment required [61].

Alternatively, the ethanol dilution method represents a durable, easily scalable, and reproducible method that provides high encapsulation efficiency of nucleic acid (>80%) into LNPs. This method is based on dissolving lipid components in ethanol, a water-miscible solvent, at specific concentrations. The alcoholic solution of lipids is then mixed at an acidic pH with an aqueous solution of mRNA using a T-shaped mixer. Mixing of the two solutions reduces the solubility of lipids (due to the dilution of ethanol), which condenses as positively charged particles that, in turn, complex with the negatively charged nucleic acid to produce nanoparticles. The resulting nanoparticles are further stabilized by another dilution with a neutral buffer to remove ethanol and neutralize the pH to obtain more stable and homogenous LNPs [25,77].

Microfluidic mixing devices have also been used for efficient and quick production of monodispersed LNPs at small scale [78]. These devices are based on injecting an alcoholic solution of lipids, which is then to be mixed with an aqueous solution using a herringbone micromixer structure built within the device. The presence of a micromixer with a microfluidic device allows rapid mixing of the two solutions and promotes the formation of small-sized LNPs. In addition, the use of the different cycle numbers of the micromixer may confirm that mixing of the solutions will lead to the production of LNPs [78].

It was reported that using a microfluidic mixing technique that employs a herringbone micromixer in preparation of siRNA–LNPs resulted in higher encapsulation efficiency (approximately 70%) of the nucleic acid compared to T-tube mixing method. However, cryo-transmission electron microscopy examination of the morphological structure of siRNA–LNPs produced by either mixing techniques revealed that both methods of mixing produced LNPs with similar structures that resemble an electron-dense core instead of the less dense aqueous core observed for liposomes. The authors found that the electron-dense core is dependent on the charge and degree of saturation of the ionizable cationic lipids used in the formulation [79,80].

## 5. Factors Affecting the In Vivo Delivery and Uptake of RNA/LNP Vaccines

### 5.1. Route of Administration

Different administration routes, such as intramuscular (IM), intradermal (ID), subcutaneous (SC), intravenous (IV), intranodal (IN), and intratumoral injections, can be used for the delivery of mRNA-based vaccines. A selection of the most appropriate administration route is an important factor that determines the safety and efficacy of vaccines. In addition, the administration route may determine the specific extracellular barriers that must be considered in the formulation of optimized LNPs [61].

In the case of cancer immunotherapy, intravenous delivery of mRNA-based cancer vaccines was found to be more suitable to stimulate a systemic immune response and, thus, produce immunity against cancer [50,81]. In addition, it was reported that intravenous and intraperitoneal immunization of mice with LNPs loaded with modified mRNA encoding firefly luciferase resulted in the highest expression in liver, but for the shortest duration (1–4 days), while intradermal and intramuscular injections of the same vaccine maintained protein production at the site of injection for 10 days [82].

On the other hand, the study conducted by Liang et al. [8] showed that intradermal immunization of rhesus macaques with a vaccine of nucleoside-modified mRNA encoding influenza H10N8 full-length hemagglutinin (HA) encapsulated in LNPs produced significantly higher hemagglutinin inhibition (HAI) titers and higher CD4+ T cell activation compared to the group of animals immunized by intramuscular injection. This superior immunogenicity produced after intradermal delivery of the vaccine was attributed to the more rapid stimulation and migration of the skin’s dendritic cells to draining lymph nodes in addition to the availability of antigens for a prolonged time at the site of injection [8].

Therefore, the skin appears to be an effective route of administration for mRNA-based vaccines as it provides direct local transfection and stimulation of dendritic cells that consequently migrate to the lymphatic system. Moreover, passive drainage of mRNA–LNPs via the lymphatic system allows direct delivery of mRNA to the lymph nodes and the resident antigen-presenting cells and T cells within [61].

To obtain effective lymphatic transport of nanoparticles, the particle size should be considered as another important factor. It was reported that nanoparticles up to 200 nm in size can enter lymphatic vessels, while larger particles can be retained at the site of injection [83,84,85]. Studies have also shown that PEGylation of LNPs may facilitate their drainage into the lymphatic system as well as their distribution and cellular uptake at the site of injection. In addition, attachment of targeting ligands to LNPs can increase their entrapment inside draining lymph nodes [86,87,88].

Therefore, formulation of optimized mRNA–LNPs that are uncharged, relatively small in size, and possess a sufficiently PEGylated outer surface are essential for the uptake of the final LNPs by the lymphatic system. These requirements were achieved in the mRNA nanoparticle system generated by Wang et al. [89]. The PEGylated lipid nanoparticle systems consisted of lipid-coated calcium phosphate nanoparticles enclosing mRNA encoding a melanoma-associated antigen. Functionalization of the LNPs was accomplished by attaching mannose as a targeting ligand to enable the preferential uptake of the final LNPs by the dendritic cells in the lymph nodes. The resulting mRNA–LNPs were less than 50 nm in size and were efficiently transported into the lymphatic system as early as 4 h after subcutaneous injection in mice. The authors postulated that the calcium phosphate core facilitated the dissolution of LNPs in the endolysosome compartment, allowing rapid release of nearly the whole dose of mRNA encoding melanoma antigens to the macrophages and dendritic cells resident in lymph nodes. The group also found that the mRNA vaccine encoding tyrosine-related protein 2 (TRP2) produced a strong cytotoxic T cell response, as well as the significant inhibition of tumor growth in a B16F10 melanoma mouse model [89].

### 5.2. Colloidal Stability of Formulations

The colloidal stability of mRNA–LNPs is another critical factor that should be monitored carefully to ensure efficacy of the administered formulation. It is well-known that mRNA nanoparticles may interact with the different bodily biological fluids. This interaction may result in adsorption of biomolecules or endogenous proteins to the surface of the nanoparticles, which leads to formation of a complex known as a bimolecular or protein corona [90]. The formation of a nanoparticle–corona complex may decrease the colloidal stability of mRNA–LNPs and lead to particle aggregation and earlier release of the mRNA load [91,92]. Therefore, characterization of LNPs should be carried out in undiluted biological fluids to determine their stability, size, and mRNA encapsulation efficiency. In this context, fluorescence correlation spectroscopy can be used to determine the degree of complexation of mRNA, while the technique of fluorescence single-particle tracking can be adopted for measuring the LNPs’ size and their extent of aggregation [91,92].

Modifications of the helper lipid composition of LNPs may also contribute to their stability in serum. For example, incorporation of cholesterol was reported to reduce the permeability and increase the rigidity of the lipid membrane and, therefore, helps to preserve the integrity of LNPs when present in serum [93]. In addition, inclusion of PEGylated lipids in the LNP composition may provide a stealth effect and improve the colloidal stability by reducing the capability of adsorption to plasma protein, thereby avoiding aggregation in the bloodstream. However, LNPs containing PEGylated lipids were reported to exhibit low cellular uptake and transfection [94]. Therefore, careful selection of PEGylated lipids is important in this case. Conventional PEG lipids, such as distearoyl-phosphatidylethanolamine (DSPE)-PEG2000, are known to remain firmly linked to LNPs and prohibit the release of the nucleic acid. Then, utilization of specific PEG lipids that possess short acyl chains (like ceramide–PEG lipids) may be advantageous to achieving high transfection efficiency because of their ability to gradually diffuse out of the LNPs, leading to the formation of destabilized LNPs that consequently release their nucleic acid contents [91,95].

The formation of a bimolecular corona complex may change the surface properties of LNPs, and these changes can be adopted to enhance and/or target delivery of the loaded content [90,96]. It is well-documented that the type and quantity of the individual proteins employed in the formation of bimolecular corona will be determined by the intrinsic properties of nanoparticles as well as their biological activity [90]. LNPs are known to interact with blood proteins (opsonins), such as immunoglobulins (IgG and IgM), fibronectin, and a number of complement proteins. These interactions will facilitate recognition and uptake of nanoparticles by the phagocytic cells and, consequently, promote their elimination [97]. PEGylation of LNPs may diminish the recognition of nanoparticles and their rapid removal by phagocytic cells. It was reported that the inclusion of PEGylated lipids lowers the tendency of nanoparticles to adsorb complement proteins and immunoglobulins and, hence, allow nanoparticles to escape the macrophage system [94]. However, accelerated removal of PEGylated nanoparticles and stronger activation of complement interactions can be encountered upon infusion of these PEGylated nanoparticles in patients having anti-PEG antibodies [94,98].

### 5.3. Incorporation of Targeting Moieties

Inclusion of antibodies or targeting ligands in mRNA–LNP systems is used to promote the disposition of mRNA in selective organs, as well as the uptake of mRNA by specific surface receptors expressed by specific immune cells, such as dendritic cells [61]. By targeting specific receptors, nanoparticles may be directed into intracellular trafficking pathways that protect the mRNA load against degradation [99]. The concept of targeting mRNA nano-vaccines towards different C-type lectin receptors, such as DEC-205 [100], CLEC9A [101], and mannose [102] receptors, can be utilized to induce the uptake of mRNA–LNPs by these specific receptors. In addition, endocytic protection of the nucleic acid from degradation and regulation of the cross presentation of dead cell-associated antigens were achieved by inclusion of targeting moieties [100,101,102].

The group of Parhiz et al. [103] investigated the binding of nucleoside-modified mRNA–LNPs to antibodies against vascular cell adhesion molecules (PECAM-1 or platelet endothelial cell adhesion molecule-1). The results revealed a 200-fold enhancement of mRNA delivery and a 25-fold elevation of protein expression in endothelial cells of the lungs after systemic administration in mice compared to the non-targeted LNPs [103].

In addition, incorporation of mannose motifs into nanoparticles led to increased transfection of mRNA mannosylated histidylated lipopolyplexes into the dendritic cells of the spleen following intravenous injection in mice [102]. Further, a modular targeting platform based on inclusion of a lipoprotein inside siRNA–LNPs was proposed by Kedmi et al. [104]. The formulated nanoparticles were designed for diverse leukocytes and to interact non-covalently with the antibody crystallizable fragment domain of a selected targeting antibody. The therapeutic efficiency of these LNPs in treating inflammatory bowel disease was demonstrated, where targeted expression of interleukin 10 was observed in inflammatory leukocytes that are only present in inflamed tissues [104,105].

Although the inclusion of targeting ligands has been shown to enhance the delivery and therapeutic efficiency of mRNA–LNPs, it should be noted that attaching targeting moieties may add complexity, cost, and regulatory difficulties to the process of manufacturing LNP systems [106]. In addition, the targeting specificity of some targeting ligands may disappear when lipid nanoparticles are exposed to biological fluids where interaction with proteins in the media and the consequent formation of protein corona takes place [107]. Therefore, a compromise between the possible clinical benefits and the complexity and cost of the targeted mRNA–LNP manufacture should be taken into consideration.

Some studies proposed that interaction of nanoparticles with opsonic blood proteins (i.e., opsonization) can be employed as a passive approach for targeting antigen-presenting cells [108,109] or to generate potent activation of dendritic cells through complement interactions in vaccine platforms [110]. A study conducted by Reddy et al. [110] demonstrated that intradermal injection of ultra-small ovalbumin nanoparticles as a vaccine platform resulted in targeting and activation of the dendritic cells residing in lymph nodes through complement interactions [110]. The authors concluded that intradermal administration of ovalbumin nanoparticles into mice may induce only humoral immunity via targeting lymph nodes and complement activation.

The differences in site-specific targeting and organ distribution after systemic administration of mRNA–LNP formulations can be attributed to the biomolecular corona and surface charge of LNPs. For example, adsorption of apolipoprotein E as a targeting ligand led to specific hepatic uptake of (si)RNA from ionizable (or near neutral) LNPs, but not from cationic LNPs [111]. On the other hand, cationic mRNA lipoplexes prepared from cationic lipid and cholesterol, for the delivery of nucleoside-modified mRNA and monophosphoryl lipid A (a TLR4 agonist), were found to accumulate primarily in the lungs transfecting their dendritic cells [112]. Unexpectedly, Kranz et al. [50] found that reducing the lipid content of mRNA lipoplexes imparted a negative charge to the nanoparticles and shifted their accumulation from the lungs to the spleen. Clinical investigation (phase I) of targeting anionic mRNA lipoplexes demonstrated accumulation of the nucleic acid in the spleen of melanoma patients [113].

### 5.4. Incorporation of Adjuvants

Enhancement of immune response towards antigens can be achieved by inclusion of certain compounds, known as adjuvants, into the vaccine formulation. The use of adjuvants in vaccine formulation allows more selective stimulation of immunological pathways to obtain antigen-specific immune responses (cell mediated or humoral) [114].

LNPs can act as adjuvants by themselves due to their lipid content. Some lipids can induce inflammation and stimulate the immune system [1]. For example, LNPs prepared with cationic lipids, such as 1,2-dioleoyl-3-trimethylammonium propane (DOTAP), for the delivery of RNAi have been reported to induce both a greater pro-inflammatory response with Th1 cytokines and activation of Toll-like receptor 4 (TLR4) in treated mice than observed with LNPs prepared using anionic or neutral lipids [115].

In addition, a pro-inflammatory response was observed in mouse skin 24 h after injection of LNPs of mRNA coding for green fluorescent protein (GFP) that were prepared with ionizable cationic lipid [1]. Therefore, activation of Toll-like receptors expressed on antigen presenting cells (APCs) has become the target of adjuvant incorporation to stimulate the production of cytokines which will initiate inflammation.

Different types of adjuvants that act as Toll-like receptor agonists have been evaluated. For instance, incorporation of bacterial monophosphoryl lipid A into LNPs resulted in more effective vaccines compared to vaccines prepared with nonlipid A formulations [116]. Adjuvants, such as unmethylated CpG-oligodinucleotides, were reported to enhance immune response when co-encapsulated with protein vaccines [117,118]. Moreover, small-molecule immune potentiators, developed by Wu et al. [119] as TLR agonists to induce immune activation, could be useful as adjuvants in formulating LNPs [1].

Incorporation of non-mRNA adjuvants may not be required. Mammalian cells can recognize foreign mRNA with different pattern recognition receptors such as the innate immune receptors TLR3, TLR7, and TLR8. Stimulation of these receptors will lead to transcriptional upregulation of genes encoding for pro-inflammatory cytokines, chemokines, and type 1 interferons [120]. These receptor-mediated responses will help to initiate immune responses.

## 6. Pre-Clinical and Clinical Applications of LNPs as Delivery Systems for RNA-Based Vaccines

The application of LNPs as delivery systems for RNA-based vaccines is beneficial for reaching the full potential of the vaccine as these systems serve to protect the large encapsulated nucleic acid molecule against nuclease degradation [1,121]. As delivery systems, LNPs pass through the cell membrane for cellular uptake (by endocytosis) and deliver their enclosed mRNA into the cytosol only after endosomal escape. LNPs may also affect the innate immune response and provide the mRNA vaccines with synergistic adjuvant effects [2].

The various application roles of RNA for both preventive and therapeutic uses have led to different employments of RNA-based vaccines against both infectious pathogens as well as cancer. Many research studies that have focused on LNPs as delivery systems for self-amplifying mRNA and conventional mRNA against various infectious diseases have shown robust and rapid immune stimulation in different animal species [9,27,29,30,122,123,124]. Examples of mRNA vaccines in various lipid-based formulations developed for preclinical studies against infectious diseases and cancer are summarized in Table 1.

In addition, different mRNA vaccine formulations developed for protection against infectious conditions have entered clinical studies to evaluate their effectiveness. Table 2 summarizes the status of these clinical trials. The clinical study conducted by Bahl et al. [27] and sponsored by Moderna Therapeutics is considered as the first human trial (NCT03076385) that used an LNP-formulated mRNA vaccine encoding the HA antigen of influenza H10N8. This study revealed that all 31 participants developed specific antibodies titers of ≥40 against the HA antigen of influenza H10N8 after two intramuscular immunizations (using 100 µg of the vaccine) separated by 3 week intervals, indicating good immunogenicity of the vaccine. In addition, the study showed that virus-neutralizing antibodies titers of ≥20 were present in the serum of 87% of the vaccinated participants after 43 days of vaccination. The results obtained from human trials were considered satisfactory despite low values of titers compared to those obtained from animal models [27]. Similar findings were reported by the group of Feldman et al. [125]. The work of these authors demonstrated that LNP-formulated mRNA vaccines encoding full-length HA from the H10N8 and H7N9 influenza strains were safe and able to produce robust humoral immune responses in healthy adults after intramuscular vaccination with two doses 3 weeks apart at 100 and 25 µg dose levels [125]. The authors also reported that the safety and reactogenicity profiles of both vaccines, used at doses up to 100 µg, were comparable to those obtained for licensed vaccines formulated with or without adjuvants [125].

### 6.1. RNA/LNP Vaccines against Severe Acute Respiratory Syndrome Coronavirus 2 (SARS-CoV-2) Infection

Nowadays, RNA-based vaccines have become one of the most effective vaccine technologies developed to protect against the pandemic due to coronavirus (COVID-19), which emerged in December 2019 due to infection by severe acute respiratory syndrome coronavirus 2 (SARS-CoV-2) [122].

Various pre-clinical studies [130,131,132,133,134] were conducted to evaluate the efficacy and immunogenicity of vaccines based on LNP–mRNA encoding for the SARS-CoV-2 spike protein or the spike receptor binding domain. For example, immunization of mice with self-amplifying RNA encoding the virus spike protein encapsulated in LNP formulation produced markedly high SARS-CoV-2-specific antibodies and induced a robust cellular immunity response compared to electroporated pDNA vaccine. These observations were attributed to the nature of the LNP formulation that was used [132].

Another preclinical study evaluated the immunogenicity of nucleoside-modified mRNA encoding the full-length SARS-CoV-2 spike protein or the spike receptor binding domain in mice. It was observed that the two vaccines induced strong T and B cell responses in addition to potent antibody responses after a single dose [130]. Further, an mRNA vaccine (mRNA-1273) encoding the spike protein of SARS-CoV-2 in the LNP formulation was developed by Moderna Therapeutics, Cambridge, MA, USA. Immunization of non-human primates with this vaccine produced high neutralizing activity and remarkably elevated S-protein-specific antibodies [135].

The results obtained from preclinical studies on mRNA vaccines formulated in LNPs were promising in providing a vaccine solution for COVID-19, and enabled the transfer of RNA-based vaccines to the level of clinical studies. Moderna mRNA-1273 is considered to be the first vaccine that entered phase I clinical studies (ClinicalTrials.gov identifier NCT04283461) only 42 days after identification of the genetic sequence of SARS-CoV-2 [122]. The manufacturing company recently announced this vaccine to be 94% effective based on the first interim analysis of phase III clinical studies (ClinicalTrials.gov identifier NCT04470427) [136].

Different institutions and pharmaceutical manufacturers are exploiting different LNPs as platform technologies to develop RNA-based vaccines against COVID-19. For example, the mRNA vaccine (BNT162) developed by BioNTech (Mainz, Germany) in collaboration with Pfizer (New York, NY, United States) was designed to comprise four different mRNA formats that target antigens of the S-protein and receptor binding domain and formulated as an LNP formulation [122,137,138]. As of 9 November 2020, BioNTech (Mainz, Germany) and Pfizer (New York, NY, United States) reported that the BNT162 vaccine was more than 90% effective against COVID-19 based on the first interim efficacy analysis from the phase III clinical studies (ClinicalTrials.gov Identifier: NCT04368728) [139].

Overall, the use of LNPs to formulate mRNA-based vaccines can be considered a promising approach for vaccination against SARS-CoV-2. These lipid-based formulations may be rapidly manufactured and, hence, accelerate vaccine development. Different mRNA-based vaccines against COVID-19 that are formulated in LNPs were shown to be safe and immunogenic in various clinical trials. The data obtained from animal studies revealed that LNP–mRNA vaccines induced a strong neutralizing antibody response and provided high protection against SARS-CoV-2.

### 6.2. RNA/LNP Vaccines against Influenza Virus Infection

Influenza vaccines based on mRNA are one of the most extensively studied mRNA vaccines because of the ease of evaluating their efficacy in small animals in addition to the possibility of measuring induced T and B cell responses. It was reported that a self-amplifying mRNA vaccine can be produced within a short period of time once the genetic sequence coding of influenza hemagglutinin (HA) antigen is identified [4]. Based on this, a self-amplifying mRNA vaccine against a strain of H7N9 influenza in China was produced (encapsulated in LNPs) within 8 days after identification of the gene sequence of HA antigen and demonstrated to be immunogenic in mice [4].

Lindgren et al. [28] also developed modified non-replicating mRNA vaccine encoding HA of a pandemic H10N8 influenza strain in an LNP formulation. Intramuscular and intradermal immunization of rhesus macaques with this vaccine produced an expansion of B cell responses along with the formation of germinal centers (GCs) in draining lymph nodes after each vaccination. In addition, an increase in the level of H10-specific T follicular helper cells was observed and correlated with high-avidity antibody responses, which take place after seasonal influenza vaccination in humans, indicating seroconversion [28].

A universal influenza vaccine was developed using an mRNA–LNP formulation to induce a potent immune response against conserved epitopes (chimeric and headless hemagglutinin structures) of different virus strains. This universal influenza vaccine raised antibodies against the stalk domain of hemagglutinin and showed good protection of animals against a wide range of influenza viruses [140]. Another broadly protecting influenza vaccine was produced and evaluated by Pardi et al. [29] in which nucleoside-modified mRNA–LNPs that express the full-length influenza virus HA were used. A single immunization with the formulated vaccine resulted in HA stalk-specific antibody responses in different animal models (mice, rabbits, and ferrets) and, hence, provided protection against homologous, heterologous, and heterosubtypic influenza virus infections in mice.

Further, LNPs were utilized for delivery of a modified mRNA vaccine encoding the HA of either H10N8 or H7N9 influenza strains [27]. The formulated vaccine produced robust, rapid, and long-lasting immune responses in mice, ferrets, and cynomolgus monkeys.

### 6.3. RNA/LNP Vaccines against Rabies Virus Infection

LNPs were employed to formulate mRNA vaccines against rabies virus infection. A sequence-optimized, unmodified mRNA vaccine, encoding rabies virus glycoprotein (RABV-G) was developed by Lutz et al. [9]. A single immunization of cynomolgus monkeys with this vaccine resulted in induction of the virus neutralization titers which exceeded the reference values set by the World Health Organization (0.5 IU/mL) to correlate with protection in humans. These titers were dose-dependent and were further enhanced by a 20 fold increase following the second immunization of the animals performed at day 28. The authors noticed that the protection against rabies remained stable during the observation period of 1 year [9].

### 6.4. RNA/LNP Vaccines against Zika Virus Infection

Utilization of mRNA/LNPs vaccines for protection against Zika virus has been described in the literature by different research groups [30,31,123]. The group of Pardi et al. [30] have shown that single intradermal vaccinations of mice (30 µg dose) or rhesus macaques (50 µg dose) with nucleoside-modified mRNA/LNPs vaccine encoding the pre-membrane and envelope (prM–E) glycoprotein of Zika virus resulted in potent and persistent protective immunity in both animal models with induction of anti-Zika virus neutralizing antibodies. Similar results were reported by Richner et al. [123] upon intramuscular vaccination of mice with two 10 µg doses of modified mRNA/LNPs vaccine encoding the prM–E glycoprotein of Zika virus. Further, the groups of Richner et al. [31,123] engineered an mRNA/LNP vaccine with mutations destroying the conserved fusion-loop epitope of the E protein. This mutant mRNA vaccine was found to be protective against Zika virus infection, and reduced production of antibodies enhancing dengue virus infection (which is closely related to Zika virus infection) in both cell culture and mice. The same vaccine was evaluated for its ability to protect the fetus against congenital malformation that may occur during pregnancy due to the transmission of Zika virus. The authors reported that two immunizations with the vaccine protected the pregnant mouse against maternal, placental, and fetal infection by Zika virus [31].

Furthermore, vaccines based on mRNAs can be designed to deliver multiple mRNAs encoding different antigens in order to produce immunity against multiple pathogens or against different antigens of the same infecting pathogen after a single immunization. These multivalent mRNAs encoding different antigens are particularly useful for stimulating different immunity responses or to target antigens expressed in multiple life cycles of the infecting pathogen [2]. For example, the work of John et al. [32] described the production of LNPs encapsulating nucleoside-modified mRNAs encoding the five different subunits of the human cytomegalovirus (CMV) pentameric protein complex and glycoprotein B (gB). The produced vaccine was efficiently delivered in vivo and resulted in potent immune responses and broadly neutralizing antibodies in both mice and non-human primates after intramuscular immunization. The authors also formulated an additional LNP/mRNA vaccine encoding the immunodominant CMV T cell antigen, pp65. Administration of this conventional vaccine with the pentameric protein and gB vaccine resulted in multi-antigenic or broad T cell responses and did not interfere with the levels of antibodies produced by vaccinating mice with the multivalent pentameric protein [32]. The mRNA vaccine expressing the pentameric proteins of human CMV has entered the clinical evaluation step and is currently in phase I clinical trials [2].

### 6.5. RNA/LNP Vaccines against Cancer

Cancer vaccines act either as a prophylactic (to prevent infections by cancer-causing viruses) or therapeutic (to treat existing cancer). The first therapeutic cancer vaccine, Sipuleucel-T (provenge) was approved in 2010 for treatment of prostate cancer [141,142]. The clinical benefits of cancer vaccines to decrease the recurrence of cancer and to improve the overall survival of patients have been established in different studies [141,143].

Various antigens, such as tumor-associated antigens (TAAs) and tumor-specific antigens (TSAs), can be encoded into cancer vaccines. TAAs comprise proteins that are overexpressed in cancer cells but also present in normal cells. e TSAs are expressed only in tumor cells and are derived from oncogenic proteins of viruses or from proteins produced upon gene mutations or rearrangements [23]. In addition, mutations in tumor cells during progression and carcinogenesis may lead to the production of altered proteins termed neoantigens. These types of proteins can be recognized by the analysis of genetic mutations in an individual cancer cell. Utilization of neoantigens may allow the production of personalized neoepitope cancer vaccines that could be advantageous in enhancing tolerance and limiting normal tissue toxicity, as well as to improve antitumor immune response compared to conventional cancer vaccines [23,142]. Personalized RNA mutanome vaccines [144] and personalized peptide vaccines [145] are examples of personalized vaccines that have shown promising results in phase I clinical trials.

The safety, immunogenicity, and tolerability of the first personalized IVAC MUTANOME (BioNTech RNA Pharmaceuticals GmbH), which is a poly-neoepitope-coding RNA vaccine, have been evaluated in phase I clinical trials (ClinicalTrials.gov identifier: NCT02035956) targeting mutant neoantigens for the treatment of patients with melanoma. A strong immune response against the vaccine antigens was observed. In addition, T cell response was generated against 60% of the 125 selected neoepitopes with no adverse drug reactions, indicating good tolerability of the vaccine by enrolled patients [146].

The possibility of obtaining RNA from a tumor sample, to produce patient-specific antigens and to then formulate a personalized vaccine is considered among the advantages of mRNA cancer vaccines compared to other types of vaccines. In addition, signals provided through the Toll-like receptors TLR3, TLR7, and TLR8 may reflect the adjuvanticity of mRNA to enhance immune response [23]. Furthermore, mRNA vaccines do not possess the risk of infection, and their manufacture is rapid, scalable, and inexpensive [14,142,147].

mRNA vaccines stimulate a specific immune response when the encoded antigen is translated to proteins in the cytosol of antigen-presenting cells or APCs (either dendritic cells (DCs) or macrophages). The expressed proteins are presented on major histocompatibility complex (MHC) class I molecules to CD8+ T cells, stimulating the cellular response. Induction of supportive CD4+ T helper cell response, which is crucial in cancer immunotherapy, may take place by fusion of the mRNA-encoded antigen to MHC class II trafficking signals derived from lysosomal proteins [23,147,148,149,150]. Therefore, mRNA vaccines can be developed to encode cancer-specific antigens to produce a specific T cells immune response against tumor cells [25].

Clinical trials and in vivo administration of personalized mRNA cancer vaccines utilize safe and biocompatible nanoparticle systems, such as lipid nanoparticles and liposomes, to formulate and optimize mRNA delivery [25,142]. The application of nano-particulate systems as carriers for mRNA cancer vaccines provides advantages in protecting the mRNA from degradation in addition to the enhancement of antitumor responses by the possibilities of co-delivery of the vaccine with an adjuvant and the utilization of ligands to target dendritic cells. In addition, nanoparticulate systems may provide the possibility to control the release and distribution of the vaccine [44,142].

Personalized mRNA cancer vaccines encoding different antigens have been formulated in lipid nanosystems and have already entered clinical studies (NCT03897881, NCT02316457, NCT03313778, NCT03480152, NCT03323398) [23,25]. The successful application of LNPs to encapsulate personalized mRNA vaccines has been described for the treatment of patients with melanoma [144]. All patients demonstrated T cell responses against the neoepitopes of the vaccine. In addition, highly reduced rates of metastases were observed in two subjects after the start of vaccination, which led to the enhanced survival of patients [144].

In order to induce a strong cytotoxic CD8 T cell response, the group of Oberli et al. [44] developed LNPs for the delivery of an mRNA vaccine encoding the model immunology protein, ovalbumin (OVA). The authors identified an optimum formulation that contains an ionizable lipid (cKK-E12) and an additive (sodium lauryl sulfate). The optimal formulation showed increased T cell response upon reducing the molar ratio of cKK-E12 from 35% to 10%. Immunization of model mice with transgenic OVA-expressing tumor or with aggressive B16F10 melanoma using the formulated mRNA vaccine encoding the corresponding antigens resulted in strong CD8 T cell immunity activation in addition to slow tumor growth, shrinkage of tumor and, consequently, extended survival of treated mice [44].

Enhancement of the LNP potency through delivery of their encapsulated mRNA can be achieved by attachment of different moieties to the surface of LNPs for targeting specific receptors on the surface of immune cells. Alternatively, co-administration of LNPs with adjuvants may enhance the stimulation of immune response [1,47,112]. For instance, incorporation of TLR4 agonist (lipopolysaccharide; LPS) in LNPs reduced tumor growth and provided longer survival in mice with B16F10 melanoma [44]. Similarly, Verbeke et al. [112] demonstrated that co-delivering the nucleoside-modified mRNA with TLR4 agonist (monophosphoryl lipid A; MPLA) inside DOTAP–cholesterol mRNA lipoplexes induced innate immunity and allowed high antigen expression in vivo. In addition, the group of Lee et al. [47] incorporated the lipopeptide tripalmitoyl-S-glyceryl cysteine (Pam3), which is a TLR1 and TLR2 agonist, as an adjuvant in LNPs encapsulating OVA mRNA. Using Pam3–LNP formulation for intramuscular immunization of mice resulted in high expression of tumor antigens with enhanced cellular immune stimulation [47].

Further to the benefits of LNPs in the delivery of mRNA cancer vaccines, LNPs can be designed to deliver mRNAs encoding cytokines to activate immunity response and kill tumor cells without causing toxicity or side effects to healthy cells [25]. The efficiency of mRNA–LNPs encoding interleukin-12 (IL-12), an example of cytokines with anticancer activity, was examined by the group of Lai et al. [151] for suppression of tumor growth in transgenic mouse models of hepatocellular carcinoma (HCC). The systemic administration of the formulated mRNA–LNPs did not result in toxicity of healthy tissues but reduced the growth of the liver tumor and increased the survival of treated mice [151].

Another strategy to increase the specificity of therapeutic mRNAs was investigated by the group of Jain et al. [45]. The authors examined the possibility of using therapeutic mRNAs to program diseased or cancerous cells to synthesize a toxic protein that will cause self-destruction of these cells without harming healthy cells. For this purpose, microRNA (miRNA) target sites were incorporated in modified mRNAs encoding toxic or apoptotic proteins like caspase or PUMA (p53 upregulated modulator of apoptosis). The presence of miRNA binding sites will allow targeting of miRNAs that are present only in healthy cells and then enable these cells to recognize and degrade the toxic mRNA. It was found that intra-tumoral administration of LNPs loaded with these miRNA–mRNA combined sequences in mice prevented the expression of toxic proteins from the mRNA of healthy cells but selectively triggered apoptosis in tumor cells without causing systemic toxicity [45].

## 7. Conclusions

RNA-based vaccines are considered a promising, highly potent, inexpensive, and scalable platform for the design of vaccines. Compared to conventional vaccines, RNA-based vaccines are more suitable for immunization during pandemics of infectious diseases due to their rapid and cost-effective production processes. The potential of using LNPs as delivery systems for mRNA-based vaccines were evaluated in many preclinical studies and proved to be effective in protecting the mRNA from extracellular degradation in addition to enabling the entry of the nucleic acid to the target cell. However, the challenges that may appear in translating the work from animal to human clinical studies have limited the number of RNA-based vaccines reaching the market in LNP formulations. As the initial approval of Onpattro^®^ and development of various nanocarriers for mRNA, the technology of LNPs has been validated to encapsulate mRNA-based vaccines and to deliver RNA therapeutics for other applications, such as gene editing and the production of therapeutic proteins. Some formulation adjustments should be carried out to obtain high efficacy of the final parenterally administered LNP. These changes should be suitable for induction of innate immunity to treat a specific infection or a disease. Generally, the work presented in this review on LNPs as delivery systems for mRNA-based vaccines will serve as a basis for building up knowledge to further improve the efficacy and tolerability of other nucleic acid products designed in LNP formulations.

## Figures and Tables

**Table 1:** mRNA vaccines in various lipid-based formulations developed for preclinical studies against infectious diseases and cancer.

Delivery Formulation	mRNA	Lipids Used	Encoded Antigen	In Vivo Animal Model	Delivery Route	Obtained Immunity Response	References
**Infectious diseases**							
LNP	Conventional, sequence-optimized	Ionizable amino lipid, phospholipid, cholesterol and a PEGylated lipid,	HA (influenza virus)	Mice, NHPs	IM	Humoral, cellular, innate	[9]
LNP	Conventional, nucleoside-modified	Ionizable lipid: DSPC: cholesterol: PEG-lipid	HA (influenza virus)	Mice, ferret, NHPs	ID, IM	Humoral, cellular, innate, protection	[6,8,27,28,29]
LNP	Conventional, nucleoside-modified	Ionizable lipid: DSPC: cholesterol: PEG-lipid	prM-E (Zika virus)	Mice, NHPs	ID, IM	Humoral, protection	[30,31]
LNP	Conventional, nucleoside-modified	Ionizable lipid: DSPC: cholesterol: PEG lipid	PC, gB, pp65 (HCMV)	Mice, NHPs	IM	Humoral, cellular	[32]
LNP	Conventional, nucleoside-modified	Ionizable lipid: DSPC: cholesterol: PEG-lipid	gp (Ebola virus)	Guinea pigs	IM	Humoral, protection	[33]
LNP	Conventional, nucleoside-modified	Ionizable cationic lipid (Acuitas Therapeutics)/phosphatidylcholine/cholesterol/ polyethylene glycol/lipid	Env (HIV)	Mice, NHPs	ID	Humoral, cellular	[6]
Liposomes	Conventional, unmodified	Cholesterol/dipalmitoyl phosphatidylcholine/phosphatidylserine	Nucleoprotein (influenza virus)	Mice	SC	Cellular	[34]
CNE, LNP, MDNP	Self-amplifying	Squalene, DOTAP, sorbitan trioleate and polysorbate 80	HA (influenza virus)	Mice, ferrets	IM	Humoral, cellular, protection	[4,35,36]
CNE	Self-amplifying	Squalene, Span 85, DOTAP	gp140 (HIV)	Mice, rabbit, NHPs	IM	Humoral, cellular	[37,38]
CNE	Self-amplifying	Squalene, Span 85, DOTAP	gB, pp65-IE1 (HCMV)	NHPs	IM	Humoral, cellular	[38]
CNE	Self-amplifying	Squalene, DOTAP, sorbitan trioleate and polysorbate 80	SLOdm, BP-2a (streptococci)	Mice	IM	Humoral, protection	[39]
CNE	Self-amplifying	Squalene, DOTAP, sorbitan trioleate and polysorbate 80	PMIF (malaria)	Mice	IM	Humoral, cellular, protection	[40]
CNE, LNP	Self-amplifying	DSPC, cholesterol, DMG PEG 2000, DLinDMA	F (RSV)	Mice, cotton rats	IM	Humoral, cellular, protection	[38,41]
LNP	Self-amplifying	-	gp (rabies), gH/gL (HCMV)	Mice	IM	Humoral	[3]
LNP	Self-amplifying	DLinDMA: DSPC: DMG PEG 2000: cholesterol	NP, M1 (influenza virus)	Mice	IM	Humoral, cellular, protection	[10]
MDNP, NLC	Self-amplifying	Modified dendrimer and DMG PEG 2000	prM-E (Zika virus)	Mice, guinea pigs	IM	Humoral	[42,43]
MDNP	Self-amplifying	Modified dendrimer and 1 DMG PEG 2000	gp (Ebola virus)	Mice	IM	Humoral, cellular, protection	[36]
MDNP	Self-amplifying	Modified dendrimer and DMG PEG 2000	Six antigens (Toxoplasma gondii)	Mice	IM	Protection	[36]
**Cancer immunotherapy**							
LNP	Conventional, nucleoside-modified or Conventional, unmodified		Ovalbumin (OVA) or tumor-associated antigens TRP2 and gp100	Mice	SC		[44]
LNP	Conventional, nucleoside-modified	Ionizable lipid: DSPC: cholesterol: PEG-lipid	Apoptic proteins, Caspase or PUMA	Mice	IV		[45]
LNP	In vitro transcribed		Anti-HER2 Antibody	Mice	IV		[46]
LNP	In vitro transcribed	Ionizable lipid, cholesterol, DOPE, C16-polyethylene glycol2000 (PEG-lipid)	Ovalbumin (OVA)	Mice	IM	Cellular, innate immune response	[47]

Env, envelope; gB, glycoprotein B; gp, glycoprotein; HA, hemagglutinin; HCMV, human cytomegalovirus; i.d., intradermal; i.m., intramuscular; i.v., intravenous; LNP, lipid nanoparticle; NHP, nonhuman primate; PC, pentameric complex; prM-E, pre-membrane and envelope; s.c., subcutaneous; BP-2a, group B Streptococcus pilus 2a backbone protein; CNE, cationic nanoemulsion; F, fusion protein; M1, matrix protein 1; MDNP, modified dendrimer nanoparticle; NHP, nonhuman primate; NLC, nanostructured lipid carrier; NP, nucleoprotein; PEI, polyethylenimine; PMIF, plasmodium macrophage migration inhibitory factor; prM-E, pre-membrane and envelope; RSV, respiratory syncytial virus; SLOdm, double-mutated group A *Streptococcus* streptolysin-O; HER2, human epidermal growth factor receptor 2; DOPE, 1,2-dioleoyl-sn-glycero-3-phosphoethanolamine; DSPC, 1,2-distearoyl-sn-glycero-3-phosphocholine; DOTAP, 1,2-dioleoyloxy-3-(trimethylammonium) propane; DLinDMA, 1,2-dilinoleyloxy-n,n-dimethyl-3-aminopropane; DMG PEG 2000, 1,2-dimyristoyl-sn-glycero-3-phosphoethanolamine-N-methoxy(polyethylene glycol)-2000.

**Table 2:** mRNA vaccines that entered clinical studies against infectious diseases and cancer.

Sponsoring Manufacturer	mRNA Vaccine	Delivery System	Target	Trial Number	Stage	Status	Reference
**Infectious diseases**							
Moderna Therapeutics/National Institute of Allergy and Infectious Diseases (NIAID)	mRNA-1273 (perfusion stabilized S protein mRNA vaccine)	LNP	COVID-19	NCT04470427	Phase III	Active, not recruiting	[126]
BioNTech / Pfizer	BNT162 (3 LNP–mRNA vaccines)	LNP	COVID-19	NCT04537949	Phase III	Recruiting	[126]
CureVac	CV7202 (sequence-optimized)	LNP	Rabies	NCT03713086	Phase I	Active, not recruiting, PCD: January 2022	[127]
Moderna Therapeutics	mRNA-1440 (nucleoside-modified)	LNP	Influenza H10N8	NCT03076385	Phase I	Completed PCD: October 2018	[27,125]
Moderna Therapeutics	mRNA-1851 (nucleoside-modified)	LNP	Influenza H7N9	NCT03345043	Phase I	Active, not recruiting, PCD: February 2020	[27,125]
Moderna Therapeutics	mRNA-1653 (nucleoside-modified)	LNP	HMPV/HPIV3	NCT03392389	Phase I	Completed, PCD: July 2019	[2]
Moderna Therapeutics	mRNA-1325 (nucleoside-modified)	LNP	Zika	NCT03014089	Phase I	Completed, PCD: July 2019	[123]
Moderna Therapeutics	mRNA-1893		Zika	NCT04064905	Phase I	Active, not recruiting, PCD: February 2021	[123]
Moderna Therapeutics	mRNA-1647 and mRNA-1443 (nucleoside-modified)	LNP	HCMV	NCT03382405	Phase I	Active, not recruiting, PCD: July 2020	[2]
Moderna Therapeutics	mRNA-1388 (nucleoside-modified)	LNP	Chikungunya	NCT03325075	Phase I	Completed, PCD: November 2019	[2]
**Cancer immunotherapy**							
BioNTech RNA Pharmaceuticals GmbH	mRNA lipoplex (Lipo–MERIT)	Liposomes	TAAs (advanced melanoma)	NCT02410733	Phase I	Active, not recruiting	[128]
BioNTech AG	mRNA lipoplex (TNBC–MERIT)	Liposomes	TAAs (triple-negative breast cancer)	NCT02316457	Phase I	Active, not recruiting	[129]

HCMV, human cytomegalovirus; hMPV, human metapneumovirus; HPIV3, human parainfluenza virus type 3; LNP, lipid nanoparticle; PCD, estimated primary completion date; TAAs, tumor-associated antigens.

## Data Availability

No new data were created or analyzed in this study. Data sharing is not applicable for this article.
